# The role of anti-diabetic drugs in NAFLD. Have we found the Holy Grail? A narrative review

**DOI:** 10.1007/s00228-023-03586-1

**Published:** 2023-11-08

**Authors:** Maria Zachou, Pagona Flevari, Narjes Nasiri-Ansari, Constantinos Varytimiadis, Evangelos Kalaitzakis, Eva Kassi, Theodoros Androutsakos

**Affiliations:** 1 Gastroenterology Department, “Sismanoglio” General Hospital, 151 26, Athens, Greece; 2 Expertise Center in Rare Haematological Diseases-Haemoglobinopathies, “Laiko” General Hospital, 115 27, Athens, Greece; 3 Department of Biological Chemistry, Medical School, National and Kapodistrian University of Athens, 115 27 Athens, Greece; 4 Gastroenterology Department, “Evangelismos” General Hospital, 106 76 Athens, Greece; 5 Department of Gastroenterology, University Hospital of Heraklion, University of Crete, 715 00 Heraklion, Greece; 6 Unit of Molecular Endocrinology, Department of Biological Chemistry, Medical School, National and Kapodistrian University of Athens, 115 27 Athens, Greece; 7 Endocrine Unit, 1st Department of Propaedeutic Internal Medicine, “Laiko” Hospital, National and Kapodistrian University of Athens, 115 27, Athens, Greece; 8 Department of Pathophysiology, Medical School, National and Kapodistrian University of Athens, Mikras Asias 75, 115 27 Athens, Greece

**Keywords:** Non-alcoholic fatty liver disease, Metabolic associated fatty liver disease, Sodium-glucose transporter inhibitors, Glucagon-like peptide-1, Pioglitazone, Metformin

## Abstract

**Purpose:**

Non-alcoholic fatty liver disease (NAFLD) has become a leading cause of liver disease, affecting 30% of the global population. NAFLD prevalence is particularly high in obese individuals and patients with type 2 diabetes mellitus (T2DM). NAFLD ranges from simple fat deposition in the liver to necroinflammation and fibrosis (non-alcoholic steatohepatitis (NASH)), NASH-cirrhosis, and/or hepatocellular carcinoma. Insulin resistance plays a key role in NAFLD pathogenesis, alongside dysregulation of adipocytes, mitochondrial dysfunction, genetic factors, and changes in gut microbiota. Since insulin resistance is also a major predisposing factor of T2DM, the administration of anti-diabetic drugs for the management of NAFLD seems reasonable.

**Methods:**

In this review we provide the NAFLD-associated mechanisms of action of some of the most widely used anti-diabetic drugs, namely metformin, pioglitazone, sodium-glucose transport protein-2 inhibitors (SGLT2i), glucagon-like peptide 1 receptor analogs (GLP1 RAs), and dipeptyl-peptidase-4 inhibitors (DPP4i) and present available data regarding their use in patients with NAFLD, with and without T2DM.

**Results:**

Both metformin and DPP4i have shown rather contradictory results, while pioglitazone seems to benefit patients with NASH and is thus the only drug approved for NASH with concomitant significant liver fibrosis by all major liver societies. On the other hand, SGLT2i and GLP1 RAs seem to be beneficiary in patients with NAFLD, showing both remarkable results, with SGLT2i proving to be more efficient in the only head-to-head study so far.

**Conclusion:**

In patients with NAFLD and diabetes, pioglitazone, GLP1 RAs, and SGLT2i seem to be logical treatment options. Larger studies are needed before these drugs can be recommended for non-diabetic individuals.

## Introduction

Non-alcoholic fatty liver disease (NAFLD) has become a major health problem worldwide with an increased global prevalence of 30%, ranging from a median of 25.1% in Western Europe to 44.4% in Latin America [1, 2]. NAFLD is in fact an umbrella term, including various stages of the disease and ranging from simple liver steatosis with fat deposition in more than 5% of hepatocytes but without inflammation (non-alcoholic fatty liver (NAFL)), to necroinflammation and fibrosis (non-alcoholic steatohepatitis (NASH)), which may progress to NASH-cirrhosis, and potentially to hepatocellular carcinoma [3, 4]. NAFLD shows an impressively high prevalence in patients with metabolic syndrome (MS) and type 2 diabetes mellitus (T2DM), especially when transaminasemia is present [5]. Moreover, NASH has been associated with an increased risk of cardiovascular-related mortality, regardless of age, sex, smoking habits, the presence of hyperlipidemia and the remaining components of MS, as well as a higher incidence of various non-liver cancers, making mandatory the early and successful treatment of the disease [6–9].

The “multiple parallel-hit” model is commonly used to explain the pathogenesis and progression of NAFLD [10]. According to this theory, different amalgamations of numerous (epi)genetic and environmental factors, representing “hits”, dynamically interplay with each other, and can drive the development and progression of the disease. These “hits” include specific genetic polymorphisms and epigenetic modifications [11], features of the metabolic syndrome, such as lack of physical activity, central obesity and adipokine dysregulation [12–14], changes in gut microbiota [13], dysregulation of autophagy and mitochondrial function [15–17], endoplasmic reticulum (ER) stress [18], hepatocyte dyshomeostasis and death [19–21], as well as inflammatory and fibrotic responses [21, 22]. The hallmark of NAFLD pathogenesis seems to be insulin resistance and an increased adipocyte-like (dys)function of the hepatocytes, when the capacity of adipose tissue to store excess energy from the diet is diminished, leading to hepatic de novo lipogenesis, steatosis and consequent inflammation and fibrosis [23–25].

Even though NAFLD poses a serious threat to patients’ health, lifestyle modifications, such as a healthy and balanced diet, weight management, and increased physical activity, are the only globally approved treatment methods [26–28]. However, since this is rarely accomplished by the majority of patients, a variety of drugs, as well as a large number of natural products, due to their availability, safety, and low cost, have been used with conflicting results [29, 30]. Among them, drugs used against type 2 diabetes mellitus, namely metformin, pioglitazone, sodium-glucose transporter-2 inhibitors (SGLT2i), glucagon-like peptide 1 receptor analogs (GLP-1 RAs), and dipeptyl-peptidase-4 inhibitors (DPP4i) have been used in both diabetic and non-diabetic individuals. In this narrative review, we will discuss the mechanisms of action of these agents in NAFLD and present published data regarding their efficacy.

## Metformin

Metformin is a biguanide of herbal origin [31] that remains the first-line medical treatment used for T2DM since the 1950s. Metformin improves glycemic control, without leading to weight gain or severe hypoglycemia [32, 33]. Metformin seems to exert its actions through multiple biochemical pathways, some of which still remain unclear [31]. The drug seems to help in glucose metabolism regulation and in the suppression of the inflammatory process, which may explain its usefulness in patients with polycystic ovarian syndrome; interestingly enough, various studies have suggested a potential protective role in the development of colorectal and hepatocellular cancer [34–37].

More specifically, metformin inhibits gluconeogenesis by acting, among others, in a mitochondrial redox state affecting hepatic glucose production [38]. Furthermore, results from in vitro and in vivo studies indicate that it reduces lipid accumulation and de novo synthesis of fatty acids primarily by contributing to the activation of AMP-activated protein kinase (AMPK) in hepatocytes [39]. Metformin also seems to induce mitochondrial fatty acid β-oxidation, thus leading to lower levels of fat accumulation in the liver, even though this finding is not univocal [40–47]. Moreover, it regulates intestinal dysbiosis by reducing bacterial toxins and restoring intestinal microbiota, while it provides protection against impaired gut barrier function; all of the aforementioned have been reported to be important in NAFLD development [48, 49].

As expected, metformin has been thoroughly investigated in NAFLD, with mainly positive results. In patients with NAFLD and T2DM, metformin has shown an improvement in glycemic control and weight loss, leading to amelioration of serum transaminases and liver steatosis, even in patients with advanced fibrosis or cirrhosis [50–56]. On the other hand, a large study, including 1292 patients with new onset diabetes starting metformin treatment and being followed up for up to 2 years, showed worsening in liver fibrosis (as expressed by means of the fibrosis-4 index (FIB-4)), but improvement in the hepatic steatosis (HIS) index, further complicating the role of metformin in these patients [57].

Metformin has also been widely investigated in non-diabetic patients with NAFLD (alone or in combination with other drugs, like liraglutide, pentoxyfilline, and probiotics), showing promising results via both patients’ weight reduction and improvement of laboratory, serum, and histological findings. However, these studies are hampered by the low number of included patients and short follow-up periods [54, 58–65]. On the other hand, several other similar studies have shown contradictory results [66–68]. This discrepancy is mirrored in published meta-analyses; some favor metformin use in patients with NAFLD, while others find no beneficial effects [68–74].

As a result of these contradictory findings, recent guidelines from international societies do not recommend metformin as a specific NAFLD treatment, due to the lack of robust data [75, 76].

## Pioglitazone

Pioglitazone and rosiglitazone are the only available agents of the drug class thiazolidinediones (TZDs), which act as peroxisome proliferator-activated receptor gamma (PPARγ) agonists. Briefly, PPARγ is expressed in various tissues, playing a key role in energy balance and lipid storage, as well as in the redistribution of intra-abdominal and subcutaneous adipose tissue by promoting the accumulation of triglyceride in peripheral fat cells depots [77–79]. As a result, TZDs lessen free fatty acid levels (through adipogenesis); increase insulin sensitivity in the liver, fat, and skeletal muscle cells; increase peripheral and splanchnic glucose uptake; and decrease hepatic glucose output [80, 81].

Among TZDs, pioglitazone is the most researched agent, with impressive in vitro and in vivo results. Apart from its use in lowering serum glucose, pioglitazone seems to retard the atherosclerotic process and reduce cardiovascular events in large trials [82–86]. Moreover, pioglitazone promotes lipid storage and redistribution from visceral to subcutaneous deposits, while enhancing the differentiation of adipocytes, rising thus as a promising agent for patients with NAFLD [4].

Several animal studies have demonstrated improvement in various aspects of NAFLD with the use of pioglitazone, including amelioration of steatosis and improvement of fibrosis [87–91]. Regarding human experiments, the first large study by Sanyal et al., including 247 non-diabetic patients with biopsy-proven NASH, under pioglitazone, vitamin E, or placebo for 96 weeks, showed no statistically significant improvement of liver histology in the pioglitazone arm [94]. However, pioglitazone improved both steatosis and levels of serum transaminases, even though it also led to weight gain. In subsequent studies, pioglitazone has been constantly associated with biochemical values and histological necroinflammation improvement, with no or minimal side-effects, apart from weight gain; unfortunately, most of these studies are small and only a handful include liver biopsies (LB) prior and post-treatment [92–107] (Table 1). Of interest, the vast majority of meta-analyses have proven that pioglitazone is both safe and effective in treating patients with NASH, even in those with significant fibrosis [107–112].

**Table 1:** Important studies of pioglitazone in NAFLD

**Author (year)**	**Drugs**	**Nr of patients**	**Type of patients**	**Duration of treatment (weeks)**	**Outcome**
Belfort et al. (2006) [92]	Diet + pioglitazone vs diet + placebo	26 vs 21	IGT or T2DM, biopsy-proven NASH	26	Improvement of LFTs and necroinflammation in liver biopsy but not in fibrosis with pioglitazone
Aithal et al. (2008) [93]	Diet + exercise + pioglitazone vs diet + exercise + placebo	31 vs 30	Non-diabetic, biopsy-proven NASH	52	Improvement in metabolic parameters, LFTs, hepatocellular damage and fibrosis, but weight gain with pioglitazone.
Sanyal et al. (2010) [94]	Pioglitazone vs vitamin E vs placebo	80 vs 84 vs 83	Non-diabetic, biopsy-proven NASH	96	Statistically significant improvement of NASH only with vitamin E. Improvent of LFTs with both agents. Pioglitazone improved steatosis and inflammation but did not reach statistical significance. Weight gain with pioglitazone
Sharma et al. (2012) [95]	Pioglitazone vs pentoxifylline	29 vs 30	Biopsy-proven NASH and increased LFTs	26	Improvement of HOMA-IR, LFTs and adiponectin with both drugs. Only pioglitazone improved liver biopsy regarding inflammation
Hajiaghamohammadi et al. (2012) [96]	Pioglitazone vs metformin vs silymarin	22 vs 22 vs 22	NAFLD, non-diabetic	8	Improvement in NAFLD parameters with all drugs. Greater reduction in HOMA-IR, glucose, TG and serum insulin levels with pioglitazone
Cusi et al. (2016) [97]	Hypocaloric diet + pioglitazone vs hypocaloric diet + placebo	50 vs 51	T2DM or prediabetes, histologically confirmed NASH	78	51% resolution of NASH, improvement in individual histologic scores, reduction in hepatic TG content, improved adipose tissue, hepatic, and muscle insulin sensitivity, but greater weight gain with pioglitazone.
Yaghoubi et al. (2017) [98]	Pioglitazone vs fenofibrate vs exercise	30 vs 30 vs 30	BMI 25–35, LFTs 1–1.5 × ULN	8	Improvement in LFTs in all groups, most with pioglitazone, weight increased with pioglitazone
Ito et al. (2017) [99]	Pioglitazone vs ipragliflozin	34 vs 32	T2DM	24	Improvement of LFTs in both groups, body weight and visceral fat reduced only with ipragliflozin
Cho et al. (2020) [100]	Dapagliflozin vs pioglitazone	27 vs 26	T2DM	At least 12 weeks pioglitazone and then 24 weeks dapagliflozin or pioglitazone	Greater improvement in FLI, body weight and waist circumference with dapagliflozin
Kinoshita et al. (2020) [101]	Dapagliflozin vs pioglitazone vs glimepiride	32 vs 33 vs 33	T2DM	28	Improvement of L/S ratio and ALT with pioglitazone and dapagliflozin
Zhang et al. (2020) [102]	Liraglutide vs pioglitazone	30 vs 30	T2DM	24	Greater decrease in LFC and fetuin-A levels with liraglutide
Shypulin et al. (2021) [103]	Pioglitazone + diet vs diet	61 vs 62	Obese, non-diabetic	12	CAP measurement improvement in pioglitazone group
Yoneda et al. (2021) [104]	Pioglitazone vs tofogliflozin	19 vs 21	T2DM	24	Improvement of MRE LS and body weight increase with pioglitazone; body weight decrease with tofogliflozin; improvement of LS in both groups
Della Pepa et al. (2021) [105]	Pioglitazone vs SU	97 vs 98	T2DM under metformin	52	Improvement of LFE, HIS, and ION; HOMA-IR, VAI, and ADIPO-IR only with pioglitazone
Gastaldelli et al. (2021) [106]	Diet + pioglitazone vs Diet + placebo	30 vs 25	IGT or T2DM, biopsy proven NASH	26	In both groups improvement of steatosis, improvement of necroinflammation only with pioglitazone, weight gain with pioglitazone, and weight loss with diet only
Yoneda et al. (2022) [107]	Tofogliflozin vs pioglitazone vs tofogliflozine + pioglitazone	21 vs 17 vs 32	T2DM	24 mono- followed by 24 weeks of combined therapy	Biochemical, HbA1C, and BMI improvement in all arms, LS improvement with pioglitazone and combination, better results with combination

*ADIPO-IR* adipose tissue insulin resistance, *CAP* continued attenuation parameter, *FLI* fatty liver index, *HbA1C* glycated hemoglobin, *HIS* hepatic steatosis index, *HOMA-IR* homeostatic model assessment for insulin resistance, *IGT* impaired glucose tolerance, *ION* index of NASH, *LFC* liver fat content, *LFE* liver fat equation, *LFTs* liver function tests, *L/S ratio* liver to spleen ratio, *LS* liver stiffness, *MRE* magnetic resistance elastography, *NAFLD* non-alcoholic fatty liver disease, *NASH* non-alcoholic steatohepatitis, *SU* sulfonylureas, *T2DM* type 2 diabetes mellitus, *TG* triglycerides, *VAI* visceral adiposity index

As a result of these studies, pioglitazone, alongside vitamin E, is recommended by international liver societies for the treatment of non-alcoholic steatohepatitis with significant fibrosis [75, 76]. Unfortunately, in most countries, pioglitazone is not available for non-diabetic patients with NAFLD, while weight gain and the risk of bladder cancer make both patients and physicians reluctant to using it.

## Sodium-glucose co-transporter type-2 inhibitors

SGLT2i are glucose-lowering agents that improve glucose control while promoting weight loss and lowering serum uric acid levels. These agents have shown great advantages even in patients with no diabetes, gaining approval for use in non-diabetic patients with heart and kidney failure [113–117]. Up till now, three SGLT2i are used in Europe, namely canagliflozin, dapagliflozin, and empagliflozin, while luseogliflozin and topogliflozin are approved only in Japan, and ipragliflozin in Japan and Russia.

SGLT2i seem to be promising agents for NAFLD treatment, since they could inhibit liver steatosis via a variety of mechanisms. Treatment with SGLT2i results in decreases in both glucose and insulin levels (especially in patients with T2DM) which in turn lead to a large decrease in hepatic de novo lipid synthesis [118]. Moreover, glucagon-secreting alpha cells of pancreatic islets express SGLT2, so the use of SGLT2i leads to increased secretion and, consequently, blood levels of glucagon [118–120]. These high glucagon levels lead to stimulation of β-oxidation; as a result, a shift from carbohydrate to fatty acid metabolism is accomplished, leading to diminished liver triglyceride content [118, 121, 122]. Another beneficial action of SGLT2i is their anti-oxidant effect. Apart from their ability to reduce high glucose-induced oxidative stress, SGLT2i reduce free-radical generation, suppress pro-oxidants and upregulate anti-oxidant systems such as superoxide dismutases (SODs) and glutathione (GSH) peroxidases [123–127]. Moreover, SGLT2i improves hepatic cell endoplasmic reticulum (ER) stress in a variety of mouse models and leads to lower levels of transforming growth factor-beta (TGFb), a potent inducer of liver fibrosis [17, 128–131].

A lot of human studies have shown the favorable effects of SGLT2i treatment in NAFLD, especially in patients with T2DM [99–101, 132–157] (Table 2). In the majority of these patients, the administration of SGLT-2i has resulted in improvement of serum transaminases, as well as improvement of liver steatosis, evaluated by radiographic criteria in magnetic resonance imaging (MRI) and ultrasound (U/S), by non-invasive scores, such as AST to platelet ratio (APRI) index, NAFLD fibrosis score (NFS) and FIB-4 score, or even by LB. In some of these studies, improvement in hepatic fibrosis was found, using transient elastography (TE) or LB, even though this finding was not univocal [134, 153, 154, 156]. Unfortunately, the vast majority of these studies are limited by their small sample size and heterogeneous inclusion criteria, especially regarding the presence of NAFLD, while almost all of them include only patients with T2DM. As a result, a variety of meta-analyses have been conducted aiming to assess the true benefit of SGLT-2i in patients with NAFLD. In the largest one, comprising 9 randomized trials, with 7281 and 4088 patients in the SGLT-2i and control arms (standard of care (SOC) or placebo), respectively, the use of SGLT-2i resulted in improvement of serum transaminases, body weight, and liver fat [158–160]. Regarding NAFLD in non-diabetic patients, only a small single-center study exists, including 12 patients under dapagliflozin and 10 patients under teneligliptin, a DPP4i, for a total of 12 weeks. At the end of the intervention, serum transaminases were decreased in both groups, while in the dapagliflozin group, total body water and body fat decreased, leading to decreased total body weight [152].

**Table 2:** Important studies of SGLT2i in NAFLD

**Author (year)**	**Drugs**	**Nr of patients**	**Type of patients**	**Duration of treatment (weeks)**	**Outcome**
Bando et al. (2017) [132]	Ipragliflozin vs SOC	40 vs 22	T2DM	12	Improvement in LFTs, VFA, and L/S ratio with ipragliflozin
Seko et al. (2017) [133]	Canagliflozin vs sitagliptin	18 vs 27	T2DM	24	Significant decrease in LFTs with both drugs, not statistically significant between SGLT-2i and sitagliptin
Akuta et al. (2017) [134]	Canagliflozin	5	T2DM	24	Improvement of NAS score and LS; fibrosis improvement in 2 pts
Ito et al. (2017) [99]	Ipragliflozin vs pioglitazone	32 vs 34	T2DM	24	Improvement of L/S ratio, ALT, ferritin not statistically significant between 2 groups; ipragliflozin more weight and VFA reduction
Eriksson et al. (2018) [135]	Dapagliflozin vs OM-3CA vs both vs placebo	21 vs 20 vs 22 vs 21	T2DM	12	Reduction of LFTs, CK-18, and FGF-21 in the dapagliflozin group and liver fat in the dapagliflozin + OM-3CA group
Bajaj et al. (2018) [136]	Canagliflozin or dapagliflozin or liraglutide or sitagliptin	1325 vs 730 vs 521 vs 661	T2DM	Retrospective	SGLT2i led to lower ALT levels (especially in higher baseline values), lower weight and better HbA1C than GLP1 RAs
Kuchay et al. (2018) [137]	Empagliflozin vs SOC	22 vs 20	T2DM	20	Reduction of liver fat and ALT
Shibuya et al. (2018) [138]	Luseogliflozin vs metformin	16 vs 16	T2DM	26	Improvement in L/S ratio compared to baseline
Choi et al. (2018) [139]	Dapagliflozin + metformin vs DPP4i + metformin	50 vs 52 (all abnormal ALT)	T2DM	44.4 ± 18.4 for dapagliflozin and 50.4 ± 21.6 for DPP4	Statistically significant decrease in dapagliflozin vs DPP4
Itani and Ishihara (2018) [140]	Canagliflozin	35	T2DM	26	Improvement in ALT, ferritin, and FIB-4 at 3 and 6 months
Miyake et al. (2018) [141]	Ipragliflozin	43	T2DM	24	Reduction in LFTs, CAP, and not statistically significant reduction in fibrosis
Shimizu et al. (2019) [142]	Dapagliflozin vs SOC	33 vs 24	T2DM	24	Improvement of CAP and LS, especially for high LS at the trial beginning
Sumida et al. (2019) [143]	Luseogliflozin	40	T2DM	24	Reduction in transaminases, serum ferritin, and liver fat in MRI
Akuta et al. (2019) [144]	Canagliflozin	9	T2DM	24	Histological improvement in all patients
Yamashima et al. (2019) [145]	Ipragliflozin (18), dapagliflozin (2), tofogliflozin (1), empagliflozin (1)	22	T2DM	52 (22 pts) and 104 (15 pts)	Lower serum transaminases levels at 12 and 24 months, better CAR and shear-wave velocity at 12 months
Han et al. (2020) [146]	Ipragliflozin + metformin + pioglitazone vs metformin + pioglitazone	29 vs 15	T2DM	24	Better FLI, CAP, and NAFLD liver fat score
Kahl et al. (2020) [147]	Empagliflozin vs placebo	42 vs 42*	T2DM	24	LFC improvement only with empagliflozin
Mittag-Roussou et al. (2020) [148]	GLP-1 RAs VS SGLT-2i	39	T2DM	24	Improvement of LFTs, HbA1c, fasting plasma glucose, BMI, and LFC in both groups. Reduction of intrahepatic lipid contents only in SGLT-2 group
Cho et al. (2020) [100]	Dapagliflozin vs pioglitazone	27 vs 26	T2DM	At least 12 weeks pioglitazone and then 24 weeks dapagliflozin or pioglitazone	Greater improvement in FLI, body weight, and waist circumference in dapagliflozine
Kinoshita et al. (2020) [101]	Dapagliflozin vs pioglitazone vs glimepiride	32 vs 33 vs 33	T2DM	28	Improvement of L/S ratio and ALT levels with pioglitazone and dapagliflozin
Akuta et al. (2020) [149]	Canagliflozin	7	T2DM	24	Histopathological improvement at 24 weeks sustained to > 1 year, LFTs and ferritin better at 24 weeks
Euh et al. (2021) [150]	Dapagliflozin (58), empagliflozin (34), ipragliflozin (3), and vsSOC (except GLP1RAs)	95 vs 188	T2DM	39	Statistically significant reduction in ALT and body weight in SLT2i vs SOC
Colosimo et al. (2021) [151]	DDP4is vs GLP-1RAs vs SGLT-2i vs metformin ± sulonylureas ± glinides ± pioglitazone	104 vs 338 vs 195 vs 165	T2DM	24 and 48	Both GLP1 RAs and SGLT2i reduced BMI, HbA1c, LFTs, FLI, and FIB-4 score
Tobita et al. (2021) [152]	Dapagliflozin vs tenegliptin	12 vs 10	NAFLD, non-diabetic	12	ALT, AST and ferritin improvement in both arms, no changes in steatosis or FIB-4 index
Takahashi et al. (2021) [153]	Ipragliflozin vs SOC (except pioglitazone, GLP-1RAs)	27 vs 28	T2DM	72	Statistically significant improvement in NASH resolution and fibrosis improvement with ipragliflozin
Chehrehgosha et al. (2021) [154]	Empagliflozin vs pioglitazone or placebo	21 vs 57	T2DM	24	Improvement in CAP-assessed liver steatosis measurements, no difference vs pioglitazone for LFTs or FIB-4 score
Gaborit et al. (2021) [155]	Empagliflozin vs placebo	18 vs 16	T2DM	12	Reduction in liver fat with empagliflozin
Yoneda et al. (2021) [104]	Topogliflozin vs pioglitazone	21 vs 19	T2DM	24	Decrease of liver steatosis in both groups, body weight decrease in topogliflozin
Arai et al. (2021) [156]	Canagliflozin (29), ipragliflozin (12), tofogliflozin (6), dapagliflozin (4), luseogliflozin(4), empagliflozin (1) vsSOC	56 vs 44	T2DM	48	Decrease in CAP assessed liver steatosis, ALT, FIB-4 with SGLT-2i
Pradhan et al. (2022) [157]	GLP-1RAs vs DDP-4i vs SGLT-2i	30.291 vs 373.741 vs 41.184	T2DM	Retrospective	Lower incidence of NAFLD compared with DDP-4i, both GLP-1 RAs and SGLT-2 decreased risk of LFTs elevation

*ALT* alanine aminotransferase, *CAP* controlled attenuation parameter, *C/T* computed tomography, *DDP4i* dipeptidyl peptidase 4 inhibitors, *FIB-4* fibrosis-4 index, *FLI* fatty liver index, *GLP-1 RAs* glucagon-like peptide-1receptor analogs, *HbA1c* glycated hemoglobin, *LB* liver biopsy, *LFC* liver fat content, *LS* liver steatosis, *FLI* fatty liver index, *L/S ratio* liver to spleen ratio, *LFTs* liver function tests, *MRI* Magnetic Resonance Imaging, *NAS* score NAFLD activity score, *OM-3CA* omega-3 carboxylic acids, *RCT* randomized controlled trial, *SGLT-2i* sodium-glucose co-transporter type-2 inhibitors, *SOC* standard of care, *VFA* visceral fat area, *VS* versus

*All patients with excellent glycemic control

Overall, SGLT2i are considered very promising agents for NAFLD, both in terms of steatosis, as well as of fibrosis, especially in patients with T2DM. However larger studies are needed, mainly in non-diabetic patients.

## Glucagon like peptide-1 receptor analogues 

There are currently six GLP-1 RAs approved for T2DM treatment: liraglutide, exenatide, dulaglutide, semaglutide, lixisenatide, and albiglutide [161]. GLP-1 is an intecrin hormone secreted by intestinal L-cells after meal digestion. GLP-1 RAs’ mechanisms of action include the induction of pancreatic b-cell proliferation and reduction of lipotoxic b-cell apoptosis, leading to improved glucose-mediated insulin synthesis and secretion and the suppression of glucose-mediated glucagon release. As a result, glucose blood levels remain low, and simultaneously, hypoglycemia is avoided [162, 163]. Moreover, GLP-1 RAs increase the insulin sensitivity of hepatocytes (through AMP-activated protein kinase), reduce peripheral insulin sensitivity and increase glucose uptake by hepatocytes and muscle cells, while, by suppressing appetite and delaying gastric emptying after meal digestion, they lead to weight loss [164–166]. Regarding the effects of GLP-1RAs on the liver, studies have shown that they act directly on human hepatocytes to decrease steatosis by preventing regeneration of fat and increasing oxidation of fatty acids, thus reducing intrahepatic fat deposits and fat-derived oxidants [167, 168].

Various clinical studies have demonstrated that GLP-1RAs have a beneficial effect in patients with NAFLD, mainly in those with concomitant T2DM [50, 51, 102, 136, 148, 151, 157, 169–195] (Table 3). In most of these studies, GLP-1RAs have shown improvement in serum transaminases, weight reduction, a significant decrease in hepatic steatosis, and improvement of NASH. In some of these studies, GLP-1RAs have even shown an ability to reduce hepatic fibrosis even though results are rather contradictory [102, 171, 173, 178, 191, 195]. In one of them, including 320 patients with biopsy-proven NASH (with 230 of them having F2-F3 fibrosis), semaglutide use for 72 weeks led to NASH resolution with no significant side effects [191]. Most importantly, GLP-1RAs have proven to be safe, with mainly gastrointestinal adverse effects, like nausea, vomiting, and diarrhea, as well as asymptomatic hypoglycemia and rarely headache, mainly after the administration of higher doses of the drugs (especially liraglutide); serious adverse events seem to be extremely rare [196].

**Table 3:** Important studies for GLP1 RAs in patients with NAFLD

**Author (year)**	**Drugs**	**Nr of patients**	**Type of patients**	**Duration of treatment (weeks)**	**Outcome**
Cuthbertson et al. (2012) [169]	Exenatide or liraglutide as add-on to metformin + DDP4i	19 vs 6	T2DM	24	GLP-1 RAs add-on related to weight loss, HbA1c and intrahepatic lipid reduction
Ohki et al. (2012) [170]	Liraglutide vs sitagliptin vs pioglitazone	26 vs 36 vs 20	T2DM	Retrospective	Improvement of serum ALT, blood glucose, and HbA1c levels in all groups. AST to platelet ratio decreased with liraglutide and pioglitazone. Body weight decreased with liraglutide
Eguchi et al. (2015) [171]	Liraglutide	27	T2DM	24 – 10 pts 96 weeks	Improvement of BMI, visceral fat accumulation, LFTs, and glucose. Decreased histological inflammation as determined by NAS and stage determined by Brunt classification in 6/10 patients that continued up to 96 weeks
Armstrong et al. (2016) [172]	Liraglutide vs placebo	7 vs 7	Biopsy-proven NASH, with or without T2DM	12	Reduction of BMI, HbA1c, LDL, ALT, leptin, and adiponectin and increase of hepatic insulin sensitivity with liraglutide
Armstrong et al. (2016) [173]	Liraglutide vs placebo	26 vs 26	Overweight patients with clinical evidence of NASH	48	Resolution of definite NASH and less fibrosis progression with liraglutide
Smits et al. (2016) [174]	Liraglutide or sitagliptin or placebo (previous treatment metformin and/or sulphonylurea)	17 vs 18 vs 17	Overweight patients with T2DM	12	No reduction of LS as assessed by magnetic resonance spectroscopy and three validated formulas for fibrosis with either liraglutide or sitagliptin
Frøssing et al. (2017) [175]	Liraglutide vs placebo	48 vs 24	PCOS with BMI > 25 and/or insulin resistance	26	Reduced body weight, LFC (assessed by HMR spectroscopy), VAT (assessed by MRI), HBA1c fasting glucose, and leptin with liraglutide. No differences in glucagon or adiponectin.
Feng et al. (2017) [52]	Liraglutide vs metformin vs gliclazide	29 vs 29 vs 29	T2DM	24	Less improvement in weight loss and LFTs, reductions in intrahepatic fat content and HbA1c levels with gliclazide, and slightly better results with liraglutide vs metformin
Petit et al. (2017) [176]	Liraglutide	68	T2DM	24	Significant decrease in body weight, HbA1c, and LFC
Bouchi et al. (2017) [177]	Liraglutide + insulin vs insulin monotherapy	8 vs 9	T2DM	24	Liraglutide led to a significant reduction of VFA, liver attenuation index, and CRP and an improvement of the quality of life scores
Khoo et al. (2017) [178]	Diet + exercise vs liraglutide	12 vs 12	Obese patients with NAFLD	26	Similar weight loss and LFTs improvement
Feng et al. (2018) [51]	Liraglutide vs metformin vs gliclazide	29 vs 29 vs 27	T2DM	24	Reduction of body fat mass, body weight and AST levels with liraglutide, and metformin; blood glucose, ALT, and HbA1c levels are reduced in all treatment arms
Tian et al. (2018) [179]	Liraglutide vs metformin	52 vs 75	T2DM	12	Improvement of serum ALT and NAFLD in U/S and HbA1c in both groups, better results with liraglutide
Zhang et al. (2018) [180]	Liraglutide vs SOC	424 vs 411	T2DM		Better lipid parameters and LFTs with liraglutide
Bajaj et al. (2018) [136]	Canagliflozin or dapagliflozin or liraglutide or sitagliptin	1325 vs 730 vs 521 vs 661	T2DM	Retrospective	SGLT2i led to lower ALT levels (especially in higher baseline values), lower weight, and better HbA1c than GLP1 RAs
Cusi et al. (2018) [181]	Dulaglutide vs placebo	971 vs 528	T2DM	24	Dulaglutide significantly reduced LFTs
Yan et al. (2019) [182]	Add-on to metformin: liraglutide vs sitagliptin vs insulin glargine	24 vs 27 vs 24	T2DM	26	Statistically significant decrease of intrahepatic lipids, VAT, and body weight and improved glycemic control in liraglutide and sitagliptin groups
Khoo et al. (2019) [183]	Diet + exercise vs liraglutide	15 vs 15	Obese patients with NAFLD	26 weeks administration and 26 weeks follow-up with only advice to prevent weight gain	Same significant reduction in weight, LFF on MRI, ALT levels, and CCK-18 at 26 weeks; at 52 weeks liraglutide group significantly regained weight and increased LFF and CCK18
Mittag-Roussou et al. (2020) [148]	GLP-1 RAs vs SGLT-2i	39	T2DM	24	Improvement of LFTs, HbA1c, fasting plasma glucose, BMI, and LFC in both groups. Reduction of intrahepatic lipid contents only in SGLT-2 group
Guo et al. (2020) [184]	Liraglutide + metformin vs insulin glargine + metformin vs placebo + metformin	32 vs 32 vs 32	T2DM*	26	Greater reduction of SAT,VAT and intrahepatic liver content in metformin + liraglutide group
Makri et al. (2020) [185]	GLP-1 RA vs DDP-4i	37 vs 152	T2DM	6–18	Improvement of LS but not liver fibrosis
Vedtofte et al. (2020) [186]	Liraglutide vs placebo	37 vs 45	Overweight non-diabetic women with prior gestational diabetes mellitus	52	Reduction of CAP-assessed LFC and body weight with liraglutide
Kuchay et al. (2020) [187]	Dulaglutide vs SOC	32 vs 32	T2DM	24	Statistically significant reductions in LFC and gGT; non-significant reduction in AST, ALT, and LS
Shiomi et al. (2020) [188]	Liraglutide	55	T2DM	24	Improvement of LFTs and FIB-4 score regardless of BMI changes or obesity status
Liu et al. (2020) [189]	Exenatide vs insulin glargine	38 vs 38	T2DM	24	Greater reduction of LFC, VAT, SAT, LFTs, body weight, waist circumference, postprandial plasma glucose and LDL with exenatide
Zhang et al. (2020) [102]	Liraglutide vs pioglitazone	30 vs 30	T2DM	24	Greater decrease in LFC and fetuin-A levels with lira
Bizino et al. (2020) [190]	Liraglutide vs placebo	24 vs 26	T2DM*	26	Greater reduction in body weight and SAT with liraglutide. No reduction in hepatic fat
Newsome et al. (2021) [191]	Semaglutide (different doses) vs placebo	240 vs 80 (230 with F2/3 liver fibrosis)	Biopsy-confirmed NASH and F1-F3 liver fibrosis	72	NASH resolution with no worsening of fibrosis on high dose of semaglutide, non-statistically significant improvement in fibrosis stage on high dose of semaglutide
Flint et al. (2021) [192]	Semaglutide vs placebo	34 vs 33	NAFLD	48	Reduction in LS, LFTs, body weight, and HbA1c with semaglutide. No reduction of liver stiffness
Li et al. (2021) [193]	Liraglutide	20	Newly diagnosed overweight T2DM	12	Liraglutide induced significant weight loss, reduction of LFC and FGF21 levels
Colosimo et al. (2021) [151]	DDP4is vs GLP-1RAs vs SGLT-2is vs Metformin ± sulfonylureas ± glinides ± pioglitazone	104 vs 338 vs 195 vs 165	T2DM	24 and 48	Both GLP1 RAs and SGLT2i reduced BMI, HbA1c, LFTs, FLI, and FIB-4 score
Harreiter et al. (2021) [194]	Exenatide and dapagliflozin vs placebo and dapagliflozin	16 vs 14	T2DM	24	Reduction of LFC and improved glycemic control in exenatide group
Pradhan et al. (2022) [157]	GLP-1RAs vs DDP-4i vs SGLT-2i	30.291 vs 373.741 vs 41.184	T2DM	Retrospective	Lower incidence of NAFLD compared with DDP-4i, both GLP-1 RAs and SGLT-2i decreased the risk of LFTs elevation
Arai et al. (2022) [195]	Semaglutide	16	T2DM	24	Improvement of serum glucose levels, LFTs, body weight and CAP measurements. Improvement of FIB-4 score but not liver stiffness

*ALT* alanine aminotransferase, *AST* aspartate aminotransferase, *BMI* body-mass index, *CAP* controlled attenuation parameter, *CCK-18* caspase-cleaved cytokeratin-18, *CRP* c-reactive protein, *DDP4i* dipeptidyl peptidase 4 inhibitors, *FGF21* fibroblast growth factor-21, *FIB-4* fibrosis-4 index, *FLI* fatty liver index, *HbA1c* glycated hemoglobin, *LDL* low density lipoprotein, *LFC* liver fat content, *LFTs* liver function tests, *LFF* liver fat fraction, *LS* liver steatosis, *MRI* magnetic resonance imaging, *NAFLD* non-alcoholic fatty liver disease, *NAS* NASH activity score, *NASH* non-alcoholic steatohepatitis, *PCOS* polycystic ovary syndrome, *SAT* subcutaneous adipose tissue, *SOC* standard of care, *T2DM* type 2 diabetes mellitus, *VAT* visceral adipose tissue, *VFA* visceral fat area, *VS* versus

*Already under metformin with inadequate glycemic control

Due to the clinical effects of GLP-1RAs, a number of studies have tried to compare them with other agents used in NAFLD treatment. The largest, so far, of these studies, is a retrospective analysis from Pradhan et al., comprising almost 450,000 patients with T2DM under SGLT2i, GLP-1RAs, or DPP4i. Both SGLT2i and GLP-1RAs were associated with a lower incidence of NAFLD, with SGLT2i showing better results than GLP-1RAs, and decreased risk of transaminases elevation [157]. Likewise, in smaller trials, GLP-1RAs (mainly liraglutide) have proven to be more efficient than DPP-4i or insulin, showing similar results with SGLT2i regarding liver fat, serum transaminases, and liver fibrosis, even though SGLT2i were found to be superior in decreasing liver fat content in one study and in reducing ALT levels in another [136, 148, 151]. Interestingly enough, in a recent study comparing 2 different GLP-1RAs, semaglutide showed better results in weight loss than liraglutide, raising the question of head-to-head studies of the different GLP-1RAs regarding their liver effects [197].

Overall, GLP-1RAs could be considered a very interesting drug choice for NAFLD, especially in obese patients with T2DM and NASH. Unfortunately, same as pioglitazone and SGLT2i, GLP-1RAs are not adequately studied in non-diabetic patients and so, no universal approval for NAFLD can be obtained.

## Dipeptyl-peptidase-4 inhibitors

As already mentioned, GLP-1 and glucose-dependent insulinotropic peptide (GIP) are the two incretins that regulate glucose homeostasis and pancreatic responses after food intake [198, 199]. Both these incretins are rapidly degraded from DPP4, an enzyme found in endothelial cells in various vascular beds, making it particularly accessible to peptide substrates in the gut, stomach, kidney, and liver [200]. In the case of insulin resistance, DPP4 synthesis and extraction are accelerated leading to faster GLP1 and GIP degradation and consequently higher blood glucose levels and b-cell exhaustion [198]. Apart from improving glycemic control, DPP4i seems to reduce T2DM-induced pancreatic beta cell dysfunction and apoptosis in vitro and in pre-clinical studies and to decrease skeletal muscle cell loss, further contributing to glycemic control [201, 202]. Up till now, 5 DPP4i have been approved by the FDA, namely sitagliptin, vildagliptin, saxagliptin, linagliptin, and alogliptin, while 5 others, namely anagliptin, teneligliptin, trelagliptin, omarigliptin, and evogliptin, are approved and used in Japan and Korea.

DPP4 is highly expressed in the liver, while the expression and serum levels of DPP-4 are elevated in steatohepatitis patients, and also correlate with hepatic steatosis, fibrosis, and hepatocyte apoptosis [203, 204], making DPP4i an attractive therapeutic option for patients with NAFLD. Moreover, in mouse models, genetic ablation of DPP4 resulted in improved insulin sensitivity and liver function, while gemigliptin alleviated both liver fibrosis and mitochondrial dysfunction [203–205].

In human studies, sitagliptin is the most commonly used DPP4i, followed by vildagliptin, linagliptin, and omarigliptin [206–212]. Unfortunately, most studies are limited by the small number of included patients and have shown rather contradictory results, with others favoring the use of DPP4i in liver steatosis and others showing no positive results. Moreover, in head-to-head studies with other drugs used for T2DM, DPP4i has failed to show improvement in most of the NAFLD parameters examined [157, 213]. In one of them, Yabiku et al., included 886 people with T2DM, comparing sitagliptin with pioglitazone, metformin, and placebo; sitagliptin failed to show any improvement in liver-to-spleen ratio [213]. Likewise, in the meta-analyses made, the benefit of sitagliptin was not consistent [214–216]. As a result, the use of DPP4i for NAFLD is not recommended.

## Discussion

NAFLD poses a significant burden in modern health systems, affecting large numbers of individuals and followed by significant morbidity and mortality. Genetic and epigenetic factors have been implicated in NAFLD pathogenesis, supporting the so-called “multiple parallel-hit” model, where multiple “hits”, dynamically interplay with each other, driving the development and progression of NAFLD. A variety of different drugs and substances have been tried for NAFLD with contradictory results.

NAFLD seems to be extremely common in patients with T2DM, with a recent meta-analysis by Younossi et al., showing a global prevalence of NAFLD among patients with T2DM of 55% [217].

This high incidence of NAFLD in patients with T2DM comes as no surprise, since insulin resistance, a hallmark of T2DM, is fundamental in NAFLD pathogenesis. More specifically, in patients with NAFLD, the increased visceral adipocyte mass and the disinhibited activity of hormone-sensitive lipase in insulin, increase triglyceride hydrolysis, leading to a subsequent increase of free fatty acids (FFA) especially in portal venous blood and consequently increased FFA liver uptake. Moreover, due to the decreased glucose consumption from skeletal muscles, lipid uptake from hepatocytes is further increased [218, 219]. On the other hand, the liver shows only partial insulin resistance, since hepatic lipogenesis remains insulin-sensitive even in states of severe insulin resistance; thus, FFA influx to the liver is further increased [220, 221]. Furthermore, hyperinsulinemia decreases apolipoprotein-B synthesis and consequently very low-density lipoproteins (VLDL)-associated lipid export from liver cells, leading to hepatic triglyceride synthesis with concomitant inhibition of triglyceride secretion as very low-density lipoproteins (VLDL) [222]. This, so-called de novo lipogenesis of the liver leads to the production of toxic metabolites, like glycerol and ceramides that in turn lead to insulin resistance and a vicious cycle that further aggregates hepatic steatosis [223].

This close effect between insulin resistance and NAFLD is also depicted in the latest updates in the nomenclature of liver steatosis, where the term NAFLD was firstly changed to metabolic-dysfunction-associated fatty liver disease (MAFLD) and, later on, to metabolic-dysfunction-associated steatotic liver disease (MASLD) [224, 225]. According to these definitions, metabolic-dysfunction-associated liver disease is diagnosed in the presence of radiological signs of steatosis when either obesity or diabetes mellitus is present, while in lean individuals, two metabolic risk abnormalities are required with prediabetes being one of them [224]. Given the critical role of insulin resistance in NAFLD development, it is logical that anti-diabetic drugs have been extensively tested in patients with NAFLD. Among them, metformin, pioglitazone, SGLT2i, and GLP1 RAs demonstrate the best results.

Metformin, one of the oldest and cheapest drugs against T2DM, exerts its beneficial action by reducing lipid accumulations and de novo synthesis of fatty acids. Although multiple studies highlight its use in patients with NAFLD, leading to the improvement of body weight and of the degree of steatosis, published data regarding its benefit in NAFLD are rather contradictory. Consequently, the drug is not routinely recommended for use in patients with NAFLD. Likewise, DPP4i, though promising as therapeutic agents, have failed to show consistent results in improving liver steatosis and fibrosis, so their use for NAFLD is not recommended.

On the other hand, pioglitazone, SGLT2i, and GLP1 RAs have shown impressive results with improvement of liver fat accumulation and resolution of NASH, rising as promising agents for NAFLD; it is no wonder that pioglitazone is the only drug approved for NASH with concomitant significant liver fibrosis by all major liver societies. Regarding the other two drug classes, both have shown remarkable results, with SGLT2i proving to be more efficient in the only head-to-head study so far. Unfortunately, GLP1 RAs are not yet approved for non-diabetic patients, while SGLT2i can be used in patients with no T2DM only under the presence of heart or renal failure, urging as mandatory the conduction of extended trials with these drugs in NAFLD patients with no T2DM.

## Data Availability

Not applicable.
